# The prognostic role of magnetic resonance enterography at diagnosis in paediatric isolated ileocaecal Crohn's disease

**DOI:** 10.1002/jpn3.70407

**Published:** 2026-03-25

**Authors:** Saverio Pochesci, Luca Scarallo, Federico Rubera, Arianna Pranzetti, Marco Di Maurizio, Massimo Basile, Paolo Emilio Orlandi, Patrizia Alvisi, Paolo Lionetti

**Affiliations:** ^1^ Gastroenterology and Nutrition Unit, Meyer Children's Hospital IRCCS Florence Italy; ^2^ Department NEUROFARBA University of Florence Florence Italy; ^3^ Paediatric Gastroenterology Unit, Maggiore “CA Pizzardi” Hospital Bologna Italy; ^4^ Radiology Unit, Maggiore “CA Pizzardi” Hospital Bologna Italy; ^5^ Radiology Unit, Meyer Children's University Hospital IRCCS Florence Italy

**Keywords:** disease progression, paediatrics, terminal Ileitis, tumour necrosis factor‐alpha

## Abstract

**Objectives:**

We aimed at identifying magnetic resonance enterography (MRE) predictors of surgical intervention and anti‐tumor necrosis factor alpha (TNF‐α) initiation in a paediatric cohort of patients newly diagnosed with isolated ileocaecal (L1) Crohn's disease (CD).

**Methods:**

A longitudinal retrospective study was conducted at two Italian paediatric referral centres (‘Meyer Children's Hospital’, Florence and ‘Maggiore Hospital’, Bologna). We collected data from L1 CD patients who underwent MRE at diagnosis between January 2011 and December 2022, with a minimum follow‐up of 2 years. Univariate and multivariate Cox‐regression analyses were performed to investigate MRE predictors for the outcomes over time.

**Results:**

Thirty‐four out of 383 (8.9%) CD paediatric patients met the inclusion criteria. Fifty per cent were male; median age at diagnosis was 14.1 years (interquartile range [IQR] 11.8–15.1 years). Ten patients (29.4%) underwent surgery, and 17/34 (50%) needed treatment escalation to anti‐TNF‐α during a median follow‐up of 4.5 years (Q1–Q3 2.4–6.6 years). Maximum bowel wall thickness (BWT) was the only MRE parameter independently associated with surgery (hazard ratio [HR] 1.54, 95% confidence interval [CI] 1.02–2.37, *p* = 0.04) and anti‐TNF‐α initiation (HR 1.56, 95% CI 1.1–2.22, *p* = 0.01) over time. BWT > 8 mm was the optimal cut‐off to identify patients who required surgery (sensitivity 78%, specificity 52%, area‐under‐the‐curve 0.73, 95% CI 0.54–0.92, *p* = 0.026).

**Conclusions:**

BWT assessed at MRE performed at diagnosis was an independent predictor of surgery and treatment escalation over time in a paediatric cohort with L1 CD. BWT > 8 mm was the optimal cut‐off to identify patients at higher risk for surgery.

## Abstract (highlights)

**What is Known:**

- Isolated ileal or ileocaecal Crohn's disease (CD) is an uncommon phenotype in children, ranging from 8% to 16% of all diagnoses.
- In a recent systematic review, 36% of strictures developed in the context of isolated ileal or ileocaecal disease. magnetic resonance enterography (MRE) is effective in predicting the prognosis of patients with CD, with significant predictors including inflammatory features and bowel wall thickening.

**What is New:**

- Maximum bowel wall thickness (BWT) measured by MRE performed at diagnosis is independently associated with the need for surgical intervention after a median time of 19.5 months and with escalation to anti‐tumor necrosis factor alpha therapy after a median time of 1 year from diagnosis.
- BWT > 8 mm might be the optimal cut‐off to identify, at diagnosis, patients that, despite the limited disease localisation, are at higher risk of surgery and might potentially benefit from a ‘more aggressive’ therapeutical approach.

## 1 INTRODUCTION

Crohn's disease (CD) is a chronic, relapsing–remitting inflammatory disease of the gastrointestinal tract, which may occur in childhood and adolescence, and whose incidence is increasing worldwide, especially in countries where such diseases were previously underreported^1^, ^2^ CD is often complicated with strictures, with up to 50% of patients needing a surgical resection within 10 years since diagnosis and up to a third requiring multiple surgeries during their lifetime.^3^, ^4^, ^5^

While extensive ileocolonic disease represents the most common presentation of paediatric CD, accounting for more than 40% of diagnoses,^6^ isolated terminal ileal or ileocaecal localisation (L1) is relatively infrequent in children and adolescents, ranging from 8% to 16%^7^, ^8^ of all diagnosis. However, L1 CD is associated with high rates of stricturing complications requiring ileocecal resection (ICR).^9^, ^10^, ^11^

Although current paediatric guidelines recommend a ‘top‐down’ approach with upfront anti‐tumor necrosis factor alpha (TNF‐α) therapy for CD presenting at diagnosis with complicated or extensive forms, no specific guidance is provided for L1 localisation.^12^ Indeed, recent studies conducted in adult populations have suggested a potential advantage of early ICR over anti‐TNF‐α therapy in L1 CD^13^, ^14^ However, surgery is still rarely considered a first‐line therapeutic option in children, considering the lifelong disease course and the substantial risk of short bowel syndrome due to multiple resections in case of disease recurrence.^3^, ^15^

There is a substantial need to identify predictors of surgery to enhance personalised care in such rare and less studied CD localisation. Features observed at magnetic resonance enterography (MRE), including prestenotic intestinal dilatation and bowel wall thickness (BWT), have been associated with a higher risk of surgery.^16^, ^17^ However, their role in predicting surgical outcomes in children with L1 CD at diagnosis remains poorly explored. The aim of this study was to identify at diagnosis clinical, biochemical, and MRE predictors of ICR and escalation to anti‐TNF‐α therapy over time in a paediatric cohort with L1 CD.

## 2 METHODS

### 2.1 Ethics statement

The study was performed in accordance with the Declaration of Helsinki, Good Clinical Practice, and applicable regulatory requirements. Ethics approval for the study was obtained from each centre's Research Ethics Board. Children and their parents or legal guardians provided informed assent and consent for enrolment.

### 2.2 Study design and population

This was a retrospective longitudinal study conducted at two national referral centres for paediatric Inflammatory Bowel Disease (Meyer Children's Hospital, Florence, Italy, and Maggiore Hospital, Bologna, Italy). We identified all children and young people diagnosed at our centres between January 2011 and December 2022. Inclusion criteria comprehended: age <17 years at diagnosis, CD diagnosis with established clinical, endoscopic, and histological criteria,^18^ disease localisation classified as L1 according to the Paris classification of paediatric Inflammatory Bowel Disease,^19^ and MRE performed within 1 month from diagnosis. Exclusion criteria were: evidence at diagnostic MRE of disease in the small bowel or in colonic segments other than the terminal ileum and/or the caecum, incomplete endoscopic assessment or MRE study at diagnosis. No patient underwent small bowel videocapsule study concomitantly. Clinical and demographic characteristics were collected, including sex, age at diagnosis, and disease behaviour using the Paris classification. Paediatric Crohn's disease activity index (PCDAI) at diagnosis and after induction treatment was retrieved from the patients’ digital and non‐electronic charts. Biochemical inflammatory markers at diagnosis were gathered, including C‐reactive protein (CRP) and faecal calprotectin (fCal), as well as serological markers such as anti‐*Saccharomyces cerevisiae* antibodies (ASCA) immunoglobulin A (IgA) and immunoglobulin G (IgG). Simple endoscopic score for Crohn's disease (SES‐CD) was collected from the reports of diagnostic endoscopies, as well as the impossibility to intubate the ileum due to the presence of a stenosis. Finally, information regarding induction treatment and dates of escalation to anti‐TNF‐α therapy were collected.

### 2.3 MRE variables

All the MRE scans were performed either on 1.5T (Achieva; Philips Medical System, Best, The Netherlands) or 3T (Achieva, Philips Medical System) scanners with a phased‐array body coil. A standardised MRE protocol was used by iterative process with two paediatric radiologists per centre, each with a minimum of 10 years of experience in the field. All MRE were performed after proper bowel distension by the administration of 70% aqueous solution of sorbitol approximately 60 min prior to the study. Each examination was performed following intravenous administration of hyoscine butylbromide as an antiperistaltic agent and a gadolinium‐based paramagnetic contrast agent. The radiologists independently produced a standardised report. One radiologist from each site (M.B. and P.E.O.), with at least 10 years’ experience in the field of paediatric inflammatory bowel disease, re‐scored all the scans, blinded to any clinical, laboratory or outcome information. Only variables reflective of inflammation were reported in the present study. Specifically, the MRE parameters of interest included BWT, disease extension, narrowest lumen, high T2 bowel wall signal, and restriction in diffusion‐weighted imaging (DWI); contrast enhancement was categorised into mild, moderate or severe; upstream dilatation was considered significant when >30 mm. The paediatric inflammatory Crohn's magnetic resonance enterography index (PICMI) was retrospectively calculated by a single expert radiologist (M.B. and P.E.O.) from each site. PICMI incorporates BWT, wall DWI, ulcerations, mesenteric oedema, and the engorgement of mesenteric vessels (comb sign), and provides a score for each intestinal segment using the formula: 3 × wall thickness ≥3 mm + 9 × DWI (0/1) + 6 × ulcers (0/1) + 6 × mesenteric oedema (0/1) + 9 × comb sign (0/1). A cumulative PICMI ≤ 10 defines ‘transmural healing’; mild, moderate, and severe inflammation have been defined by scores of 11–55, 56–120 and >120, respectively.^20^ Therapeutic decisions for the patients were made case by case after multidisciplinary review, primarily based on baseline MRE findings, and included induction with steroids plus nutritional therapy, early anti‐TNF‐α treatment, or upfront surgery in cases of significant stricturing disease, followed by post‐operative anti‐TNF‐α prophylaxis.

### 2.4 Outcomes

The primary outcome was to identify MRE predictors of ICR at follow‐up. Secondary outcomes included identification of MRE predictors of escalation to anti‐TNF‐α therapy over time.

### 2.5 Statistical analysis

Data were managed and analysed using IBM‐SPSS (v.28th) software. Categorical variables were described as frequency and percentages. Normally distributed continuous variables were presented as mean ± standard deviations (SDs). Non‐normally distributed variables were presented as medians and first–third quartile (Q1–Q3). No paired variables were analysed. The differences between categorical variables were assessed by chi‐square test or Fisher's exact test. Differences between continuous variables were assessed using Student *t*‐test or Mann–Whitney *U* test. Univariate Cox‐regression analysis was performed to identify predictors for the outcomes over time. Significant MRE predictors were included in a backward multivariate model. Receiver operating characteristic (ROC) curves were used to appraise the discriminative accuracy of BWT in predicting surgery. Cut‐off values were derived using the Youden Index, which allowed for the optimal balance between sensitivity and specificity to be identified. The identified cut‐off was used to perform a Kaplan–Meyer survival analysis, to assess progressive cumulative free‐survival rate from the outcome of interest. All statistical tests were two‐sided and *p* < 0.05 was considered the statistically significant threshold.

## 3 RESULTS

### 3.1 Patients’ characteristics

Thirty‐four out of 383 (8.9%) CD paediatric patients presented L1 phenotype. All underwent MRE examination at diagnosis and met the inclusion criteria. 50% were male; median age at diagnosis was 14.1 years (Q1–Q3 11.8–15.1 years). Median follow‐up was 4.5 years (Q1–Q3 2.4–6.6 years). Table 1 summarises patients’ characteristics at baseline. Median PCDAI at diagnosis was 27.5 (Q1–Q3 20–35), and median fCal was 500 mg/kg (Q1–Q3 260–709.5 mg/kg). Median SES‐CD at diagnostic endoscopy was 6 (Q1–Q3 4.75–8); the ileo‐caecal valve (ICV) was not traversed in 4/34 (11.8%) patients due to the presence of a stricture. Table 2 summarises MRE characteristics at diagnosis. Median maximum BWT was 8 mm (Q1–Q3 6–10 mm), whereas median disease extension was 70 mm (Q1–Q3 40–120 mm). The median upstream bowel diameter was 22 mm (Q1–Q3: 17–27 mm) with 6/34 (17.6%) patients presenting significant upstream bowel dilatation (diameter > 30 mm). Median PICMI was 27 (Q1–Q3 24–30). Comparisons between baseline characteristics of patients with opposite outcomes are shown in Table S1.

**Table 1:** Baseline characteristics of 34 paediatric patients with L1 localisation at diagnosis of Crohn's disease.

Characteristics	*n* = 34
Male sex, *n* (%)	17 (50)
Median age at diagnosis, years (Q1–Q3)	14.1 (11.8–15.1)
Median follow‐up, years (Q1–Q3)	4.5 (2.4–6.6)
VEO‐IBD, *n*(%)	1 (2.9)
PCDAI, median (Q1–Q3)	27.5 (20–35)
G1, *n* (%)	7 (20.6)
P1, *n* (%)	3 (8.8)
Laboratory exams	
fCal, mg/kg median (Q1–Q3)	500 (260–709.25)
CRP, mg/dL mean (SD)	3.27 (3.35)
IgG ASCA, *n* (%)	16 (47.1)
IgA ASCA, *n* (%)	12 (35.3)
Endoscopic features	
SES‐CD, median (Q1*–*Q3)	6 (4.75–8)
ICV not intubated, *n* (%)	4 (11.8)
Induction therapy	
Oral steroids, *n* (%)	18 (52.9)
EEN, *n* (%)	18 (52.9)
CDED, *n* (%)	13 (38.2)
Anti‐TNF‐α, *n* (%)	2 (5.9)
Post‐induction remission rate (PCDAI < 10), *n* (%)	28 (82.3)
Months to anti‐TNF‐α escalation, median (Q1–Q3)	12 (7.5–22)
Months to surgery, median (Q1–Q3)	19.5 (6–27.5)
anti‐TNF‐α before surgery, *n* (%)	5/10 (50%)

Abbreviations: ASCA, anti‐*Saccharomyces cerevisiae* antibodies; CDED, Crohn disease exclusion diet; CRP, C‐reactive protein; EEN, exclusive enteral nutrition; fCal, faecal calprotectin; G1, growth delay; IBD, inflammatory bowel disease; ICV, ileo‐caecal valve; IgA, immunoglobulin A; IgG, immunoglobulin g; P1, perianal disease; PCDAI, paediatric Crohn's disease activity index; SD, standard deviation; SES‐CD, simple endoscopic score for Crohn's disease; TNF, tumour necrosis factor; VEO, very early onset.

**Table 2:** Baseline MRE characteristics of 34 paediatric patients with L1 localisation at diagnosis of Crohn's disease.

MRE characteristics	*n* = 34
Maximum BWT, mm median (Q1–Q3)	8 (6–10)
Disease extension, mm median (Q1–Q3)	70 (40–120)
Significant upstream dilatation, *n* (%)	6 (17.6)
Upstream lumen diameter, mm median (Q1–Q3)	22 (17–27)
Narrowest lumen, mm median (Q1–Q3)	5.5 (3–8)
High T2 bowel signal, *n* (%)	26 (76.5)
DWI restriction, *n* (%)	34 (100)
Contrast enhancement	
Mild‐to‐moderate, *n* (%)	15 (41.2)
Severe (%)	19 (55.9)

Abbreviations: BWT, maximum bowel wall thickness; DWI, diffusion weighted imaging; MRE, magnetic resonance enterography.

### 3.2 Outcome: ICR over time

Ten out of thirty‐four patients (29.4%) underwent ICR during follow‐up, after a median time of 19.5 months (Q1–Q3: 6–27.5 months). Five patients out of ten initiated anti‐TNF‐α therapy before undergoing surgery. PCDAI and SES‐CD at diagnosis were higher in patients who underwent surgery at follow‐up (35 [Q1–Q3 26.25–38.75] vs. 27.5 [Q1–Q3 17.5–34.5], *p* = 0.06 and 7.5 [Q1–Q3 6.75–8.25] vs. 5 [(4–7.75], *p* = 0.02, respectively). Post‐induction clinical remission rate was lower (60% vs. 91.7%, *p* = 0.05) in the same subgroup.

Regarding MRE features, high T2 bowel wall signal was less frequently reported among patients who required surgery at follow‐up (50% vs. 80.8%, *p* = 0.03). Further comparisons for the outcome can be found in Table S1. Univariate and multivariate Cox regression analysis for the outcome is shown in Table 3. At univariate analysis, BWT and PICMI were positively associated with ICR over time (hazard ratio [HR] 1.64, 95% confidence interval [CI] 1.18–2.28, *p* < 0.01; and HR 1.12, 95% CI 1.02–1.24, *p* = 0.02, respectively), while post‐induction clinical remission and the narrowest lumen had a negative association (HR 0.21, 95% CI 0.06–0.77, *p* = 0.02 and HR 0.71, 95% CI 0.51–0.99, *p* = 0.04). High T2 bowel wall signal was negatively associated with surgery, although not reaching the threshold for statistical significance (HR 0.31, 95% CI 0.09–1.1, *p* = 0.07).

**Table 3:** Univariate and multivariate Cox regression analysis for the outcome ‘ileo‐caecal resection over time’.

Variables	Univariate analysis HR (95% CI)	*p*‐Value	Multivariate analysis HR (95% CI)	*p*‐Value
ICR (*n* = 10)				
Sex (M vs. F)	0.71 (0.18–2.85)	0.63		
PCDAI	1.12 (1.01–1.23)	0.26		
SES‐CD	1.04 (0.94–1.16)	0.45		
ICV not intubated	1.08 (0.12–8.97)	0.94		
Anti‐TNF‐α before surgery	0.29 (0.07–1.27)	0.1		
PCDAI < 10 post‐induction	0.21 (0.06–0.77)	**0.02**		
BWT	1.64 (1.18–2.28)	**<0.01**	1.54 (1.02–2.37)	**0.04**
Narrowest lumen	0.71 (0.51–0.99)	**0.04**	0.67 (0.44–1.02)	*0.06*
Upstream bowel diameter	1.06 (0.97–1.16)	0.17		
Disease extension	1.01 (0.99–1.02)	0.25		
>30 mm upstream dilatation (yes vs. no)	4.01 (0.96–19.78)	0.06		
Contrast enhancement (mild‐to‐moderate vs. severe)	0.36 (0.74–1.74)	0.2		
High T2 bowel signal (yes vs. no)	0.31 (0.09–1.1)	0.07		
PICMI	1.12 (1.02–1.24)	**0.02**	1.1 (0.98–1.23)	0.1
Anti‐TNF‐α escalation (*n* = 17)				
Sex (M vs. F)	0.38 (0.14–1.04)	0.06		
ICV not intubated	3.45 (0.74–16.04)	0.11		
PCDAI	1.01 (0.96–1.05)	0.81		
BWT	1.45 (1.09–1.93)	**0.01**	1.56 (1.1–2.22)	**0.01**
Narrowest lumen	0.96 (0.79–1.17)	0.67		
Upstream bowel diameter	1.05 (0.98–1.12)	0.14		
Disease extension	1.01 (0.99–1.02)	0.4		
>30 mm upstream dilatation (yes vs. no)	1.7 (0.58–5)	0.33		
Contrast enhancement (mild‐to‐moderate vs. severe)	1.44 (0.52–4.01)	0.48		
High T2 bowel signal (yes vs. no)	0.25 (0.44–3.57)	0.67		
PICMI	1 (0.93–1.08)	0.96	0.93 (0.83–1.03)	0.16

*Note*: Bold values indicate statistically significant.

Abbreviations: BWT, maximum bowel wall thickness; CI, confidence interval; F, female; HR, hazard ratio; ICV, ileo‐caecal valve; M, male; PCDAI, paediatric Crohn's disease activity index; PICMI: paediatric inflammatory Crohn's magnetic resonance enterography index; SES‐CD, simple endoscopic score for Crohn's disease; TNF, tumour necrosis factor.

**Figure 1:**
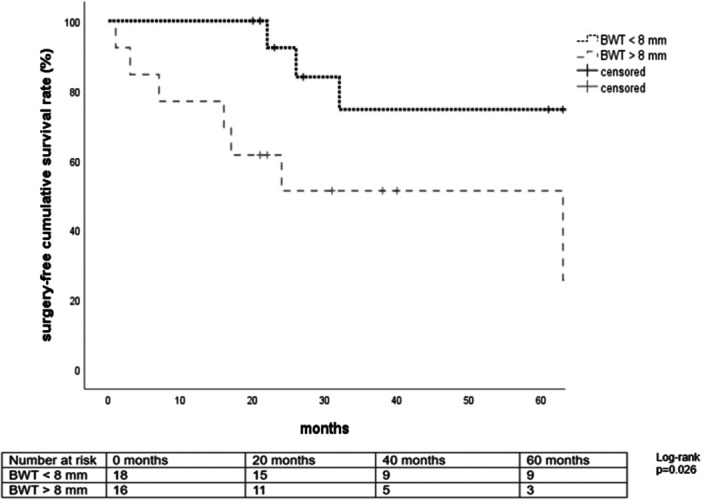
Kaplan–Meier survival analysis for ileocaecal resection with population stratified after the identified optimal cut‐off of maximum bowel wall thickness >8 mm. Number at risk can be found in the table below the figure (log‐rank *p* = 0.026). BWT, bowel wall thickness.

In multivariate analysis, BWT was the only MRE variable independently associated with surgery over time (HR 1.54, 95% CI 1.02–2.32, *p* = 0.04). An area‐under‐the‐curve of 0.73 (95% CI 0.54–0.92, *p* = 0.02) was identified when appraising the discriminative ability of BWT in predicting surgery (Figure S1). BWT > 8 mm was identified as the best cut‐off to discriminate patients who required ICR over time (sensitivity 78%, specificity 52). When stratifying the cohort by the above‐mentioned cut‐off, patients with BWT ≥ 8 mm at diagnosis had a significantly lower surgery‐free survival (*p* = 0.026) (Figure 1). Narrowest lumen and PICMI were still associated with the need for surgery over time at multivariate analysis, although not reaching the threshold of statistical significance (HR 0.67, 95% CI 0.44–1.02, *p* = 0.06 and HR 1.1, 95% CI 0.98–1.23, *p* = 0.1, respectively). Interestingly, anti‐TNF‐α therapy and time to escalation to anti‐TNF‐α therapy were not significantly associated with a lower risk of surgery over time (HR 0.21, 95% CI 0.03–1.66, *p* = 0.14 and HR 0.92, 95% CI 0.83–1.02, *p* = 0.11, respectively). Only one patient out of four (25%) with unexplored ileum at diagnostic endoscopy because of a stricture underwent surgery over time (HR 1.08, 95% CI 0.12–8.97, *p* = 0.94).

### 3.3 Outcome: Escalation to anti‐TNF‐α therapy over time

Seventeen (50%) patients needed escalation to anti‐TNF‐α after a median time of 12 months (Q1–Q3 7.5–22 months). No differences were observed when comparing the population for the outcome (Table 3). BWT was the only predictor identified and confirmed at multivariate analysis (HR 1.56, 95% CI 1.1–2.22, *p * = 0.013). Conversely, PICMI was not associated with the need for anti‐TNF‐α escalation (HR 0.93, 95% CI 0.83–1.03, *p* = 0.16). No other clinical, biochemical, endoscopic, or radiological variable was a significant predictor for the outcome (Table 3). Figure S2 shows representative MRE scans of one of the patients in our cohort.

## 4 DISCUSSION

In our cohort of children newly diagnosed with L1 CD, maximum BWT was the only MRE variable independently associated with surgery and anti‐TNF‐α escalation over time. A cut‐off >8 mm was significantly associated with reduced surgery‐free survival.

Isolated terminal ileal or ileocaecal disease represents a relatively uncommon presentation of paediatric CD, accounting for approximately 8%–16% of newly diagnosed cases.^8^, ^9^ In this disease phenotype, a European multicentre study reported a stricture rate of 13.1% over a 3‐year follow‐up period, a significantly higher incidence compared to colonic involvement, thereby underscoring the notable risk of complications despite the limited disease extent at presentation.^21^

Although current paediatric guidelines classify extensive ileocolonic disease as ‘high risk,’ warranting a top‐down approach with upfront anti‐TNF‐α therapy, there is no specific recommendation for more limited disease extension such as L1, despite its association with unfavourable outcomes in a substantial proportion of cases.^12^ Indeed, defining optimal management strategies for L1 CD remains a clinical challenge in both adult and paediatric patients. While early surgical intervention has been associated with a more favourable long‐term disease course,^13^ the potential need for repeated surgeries and the consequent risk of short bowel syndrome must be carefully considered. Therefore, there is a clear need to identify prognostic markers at diagnosis to guide personalised therapeutic approaches.

The predictive role of MRE features has been explored in previous retrospective paediatric studies.^16^, ^17^, ^22^, ^23^ Debnath et al. identified a positive association between the need for surgery and a ratio of pre‐stenotic dilation to the narrowest segment in a cohort of children with L1 CD.^22^ However, over 30% of patients in this cohort exhibited concomitant penetrating disease, which may have contributed to the high surgery rate (70%) reported; moreover, MRE was not performed at the diagnosis of CD but over the disease course. Similarly, Rosenbaum et al. reported significantly higher BWT at MRE performed within 3 months prior to surgery in children who eventually underwent resection.^16^ Yet, MRE was not performed at the time of diagnosis, and the study did not focus exclusively on L1 disease.

An expert panel from the Society of Abdominal Radiology's Crohn's Disease‐Focused Group defined ‘luminal narrowing’ as a segment of CD with unequivocal upstream dilatation (≥3 cm).^24^ Pre‐stenotic dilatation may indeed reflect stricture severity and was associated with the degree of histological fibrosis.^25^ In our cohort, however, upstream dilatation was not significantly associated with long‐term surgical risk. This finding could be partly attributed to the low proportion of patients exhibiting marked (>30 mm) dilatation at diagnosis.

It is worth noting that a significant proportion of our cohort presented inflammatory features at MRE, such as post‐contrast enhancement or high T2 bowel wall signal, with a consequent higher probability of responding to pharmacological anti‐inflammatory treatment. It may be speculated that this also accounts for the inverse association between high T2 bowel signal at diagnosis and the need for surgical intervention over time. A similar conclusion was proposed by Aloi et al., who demonstrated significant differences in inflammatory MRE findings, such as post‐contrast enhancement, mesenteric fat proliferation, and lymphadenopathy, between treatment responders and non‐responders.^23^

On the other hand, although the exact contribution of pre‐existing fibrosis—less responsive to medical therapy—to bowel wall thickening cannot be precisely quantified, it is plausible that the fibrotic component increases in parallel with wall thickness together with inflammatory oedema. This, in turn, may explain the observed association between greater BWT and a higher likelihood of requiring surgery.

In our study, we also investigated the prognostic value at diagnosis of the recently validated PICMI score. In a recent retrospective paediatric study, higher PICMI at diagnosis predicted escalation to biologic treatment. Indeed, their population also included ileocolonic and colonic disease, which accounted for almost 70% of the cohort.^26^ Conversely, PICMI did not retain significance as a predictor in our study, with the caveat of a smaller sample size and a retrospective calculation of the score. Moreover, PICMI assigns substantial weight to penetrating complications, which were absent in our cohort.

Of note, anti‐TNF‐α exposure was not significantly associated with a reduced surgical risk over time. A 2017 multicentre inception cohort study showed that early initiation of anti‐TNF‐α therapy reduced the likelihood of developing penetrating, but not stricturing, complications.^27^ Recently, 5‐year follow‐up data from the same cohort demonstrated that anti‐TNF‐α therapy commenced within 3 months of diagnosis was associated with a reduced risk of stenosis.^28^ In our cohort, however, the median time to anti‐TNF‐α initiation was 12 months, which may reflect a diminished efficacy of anti‐inflammatory treatment in reversing established fibrotic changes.

This study has several limitations. First, variability in MR image quality and bowel distension may have affected the accuracy of BWT measurements. In cases of suboptimal bowel distension, BWT cannot be reliably assessed; however, we employed a standardised protocol to promote adequate distension of the intestinal loops. Second, inter‐reader agreement for BWT and other MRE‐derived parameters was not evaluated. Given the known inter‐reader variability in the interpretation of MRE findings, the absence of reproducibility assessment may have influenced the consistency of the radiological measurements reported.

## 5 CONCLUSIONS

In a paediatric cohort with L1 CD, BWT measured by MRE at diagnosis was the only independent predictor of the need for surgical intervention and escalation to anti‐TNF‐α therapy over time. Specifically, above a cut‐off of 8 mm, each additional millimetre of BWT was associated with a 54% increase in the risk of requiring surgery. Given the retrospective design, limited sample size, and heterogeneity in clinical management, these findings should be considered exploratory. Larger, prospective studies are warranted to confirm the prognostic role of BWT at diagnosis and to better define its potential contribution to risk stratification in children with isolated ileal disease.

## CONFLICT OF INTEREST STATEMENT

The authors declare no conflicts of interest.

## Supporting information

Supplemental Table 1. Comparisons between patients with opposite outcomes. Abbreviations: PCDAI: Pediatric Crohn's Disease Activity Index; ASCA: anti‐Saccharomyces cerevisiae; fCal: fecal calprotectin: CRP: C‐reactive Protein; SES‐CD: Simple endoscopic Score for Crohn's Disease; ICV: ileo‐caecal valve; EEN: Exclusive enteral Nutrition: CDED: Crohn's Disease Exclusion diet: TNFα: Tumor Necrosis Factor alpha; BWT: bowel wall thickness; PICMI: Pediatric Inflammatory Crohn's Magnetic Resonance Enterography Index.

Supplemental Figure 1. Receiver Operating Characteristic (ROC) curve describing the ability of the maximum bowel wall thickness to predict ileocaecal resection over time (sensibility 78%, specificity 52%, AUC 0.73 (0.54‐0.92) p = 0.02).

Supplemental Figure 2. Representative scans of one of the patients in our cohort.
