# Supplementary material for: The prognostic role of magnetic resonance enterography at diagnosis in paediatric isolated ileocaecal Crohn's disease
Source: J Pediatr Gastroenterol Nutr. 2026 Mar 25;82(6):1451–7. doi: 10.1002/jpn3.70407 (PMC13238387; doi:10.1002/jpn3.70407)
Supplement: Supplementary file 1 — Supplemental Table 1. Comparisons between patients with opposite outcomes. Abbreviations: PCDAI: Pediatric Crohn's Disease Activity Index; ASCA: anti‐Saccharomyces cerevisiae; fCal: fecal calprotectin: CRP: C‐reactive Protein; SES‐CD: Simple endoscopic Score for Crohn's Disease; ICV: ileo‐caecal valve; EEN: Exclusive enteral Nutrition: CDED: Crohn's Disease Exclusion diet: TNFα: Tumor Necrosis Factor alpha; BWT: bowel wall thickness; PICMI: Pediatric Inflammatory Crohn's Magnetic Resonance Enterography Index. [file JPN3-82-1451-s003.docx]

| **Variable** | **Surgery (n=10)** | **No surgery (n=24)** | **p-value** |
| --- | --- | --- | --- |
| Male sex, n (%) | 4 (40) | 13 (54.2) | 0.46 |
| Median age at diagnosis, months (Q1-Q3) | 14 (11.1-14.8 | 14.3 (11.7-15.6) | 0.71 |
| PCDAI at diagnosis, median (Q-Q3) | 35 (26.25-38.75) | 27.5 (17.5-34.5) | 0.06 |
| G1, n (%) | 2 (20) | 5 (20.8) | 0.67 |
| P, n (%) | 1 (10) | 2 (8.3) | 1 |
| ASCA, n (%) | 4 (40) | 12 (50) | 0.71 |
| fCal, mg/kg median (Q1-Q3) | 640 (261.75-1168.75) | 500 (250-533) | 0.42 |
| CRP, mg/dl median (Q1-Q3) | 4,25 (1.75-8.4) | 1.93 (0.46-4.95) | 0.71 |
| SES-CD, median (Q1-Q3) | 7.5 (6.75-8.25) | 5 (4-7.75) | **0.02** |
| ICV not intubated, n (%) | 1 (12.5) | 3 (17.6) | 1 |
| Induction with oral steroids, n (%) | 7 (70) | 11 (45.8) | 0.27 |
| Induction with EEN, n (%) | 5 (50) | 13 (54.2) | 1 |
| Induction with CDED, n (%) | 4 (40) | 9 (37.5) | 1 |
| Induction with ATA, n (%) | 2 (20) | 0 | 0.08 |
| PCDAI<10 post-induction, n (%) | 6 (60) | 22 (91.7) | **0.05** |
| months to antiTNFα, median (Q1-Q3) | 9 (6.5-21) | 16 (9-23.5) | 0.35 |
| BWT, mm median (Q1-Q3) | 10 (7.5-11.5) | 7 (6-9) | 0.27 |
| Upstream significant dilation, n (%) | 3 (30) | 4 (16.7) | 0.39 |
| Narrowest lumen, mm median (Q1-Q3) | 5 (2.5-5.5) | 6 (3-8) | 0.11 |
| Disease extension, mm median (Q1-Q3) | 100 (62.5-150) | 65 (50-117.5) | 0.26 |
| High T2 bowel signal, n (%) | 5 (50) | 21 (80.8) | **0.03** |
| Severe contrast enhancement, n (%) | 7 (70) | 12 (50) | 0.14 |
| PICMI, median (Q1-Q3) | 28.5 (24-36) | 25.5 (24-30) | 0.95 |
| **Variable** | **anti-TNFα escalation (n=17)** | **No anti-TNFα escalation (n=17)** | **p-value** |
| Male sex, n (%) | 10 (62.5) | 7 (41.2) | 0.17 |
| Median age at diagnosis, months (Q1-Q3) | 13.7 (12.2-14.8) | 14.8 (11.1-15.9) | 0.49 |
| PCDAI at diagnosis, median (Q-Q3) | 32.5 (18.75-37) | 27.5 (22.5-32.5) | 0.12 |
| G1, n (%) | 5 (29.4) | 2 (25) | 0.23 |
| P, n (%) | 1 (5.9) | 2 (11.8) | 0.48 |
| ASCA, n (%) | 7 (41.2) | 4 (23.5) | 0.46 |
| fCal, mg/kg median (Q1-Q3) | 367 (250-500) | 500 (292.5-857) | 0.3 |
| CRP, mg/dl median (Q1-Q3) | 2.3 (1.17-6.7) | 2.1 (0.47-5) | 1 |
| SES-CD, median (Q1-Q3) | 7 (5-8) | 5 (4-8) | 0.49 |
| ICV not traversed, n (%) | 2 (11.8) | 0 | 0.48 |
| Induction with oral steroids, n (%) | 11 (64.7) | 7 (41.2) | 0.17 |
| Induction with EEN, n (%) | 10 (58.8) | 8 (47.1) | 0.3 |
| Induction with CDED, n (%) | 7 (41.2) | 6 (35.3) | 0.47 |
| PCDAI<10 post-induction, n (%) | 13 (76.5) | 15 (88.2) | 0.67 |
| BWT, mm median (Q1-Q3) | 9 (6-11) | 7 (7-9.5) | 0.21 |
| Upstream significant dilatation, n (%) | 5 (29.4) | 1 (5.9) | 0.17 |
| Narrowest lumen, mm median (Q1-Q3) | 5 (3-8) | 4 (3-6) | 1 |
| Disease extension, mm median (Q1-Q3) | 100 (40-135) | 70 (55-125) | 0.17 |
| High T2 bowel signal, n (%) | 11 (64.7) | 15 (88.2) | 0.22 |
| Severe contrast enhancement, n (%) | 11 (64.7) | 9 (52.9) | 0.49 |
| PICMI | 27 (24-30) | 30 (24-30) | 0.39 |
