# Supplementary figures and images for: The prognostic role of magnetic resonance enterography at diagnosis in paediatric isolated ileocaecal Crohn's disease
Source: J Pediatr Gastroenterol Nutr. 2026 Mar 25;82(6):1451–7. doi: 10.1002/jpn3.70407 (PMC13238387; doi:10.1002/jpn3.70407)

## Slide 1
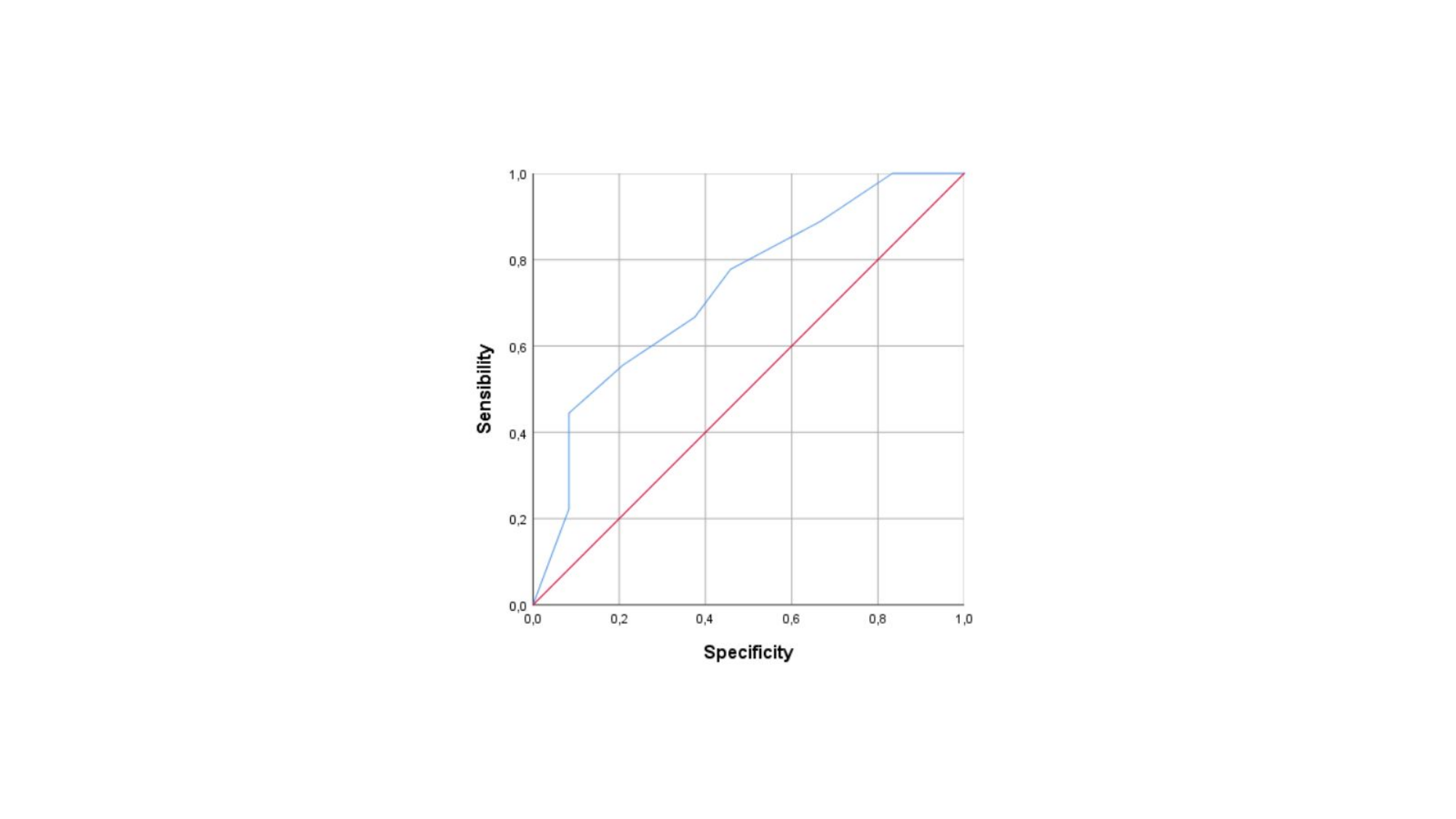

Supplement: Supplementary file 2 — Supplemental Figure 1. Receiver Operating Characteristic (ROC) curve describing the ability of the maximum bowel wall thickness to predict ileocaecal resection over time (sensibility 78%, specificity 52%, AUC 0.73 (0.54‐0.92) p = 0.02). [file JPN3-82-1451-s002.pptx]

## Slide 1
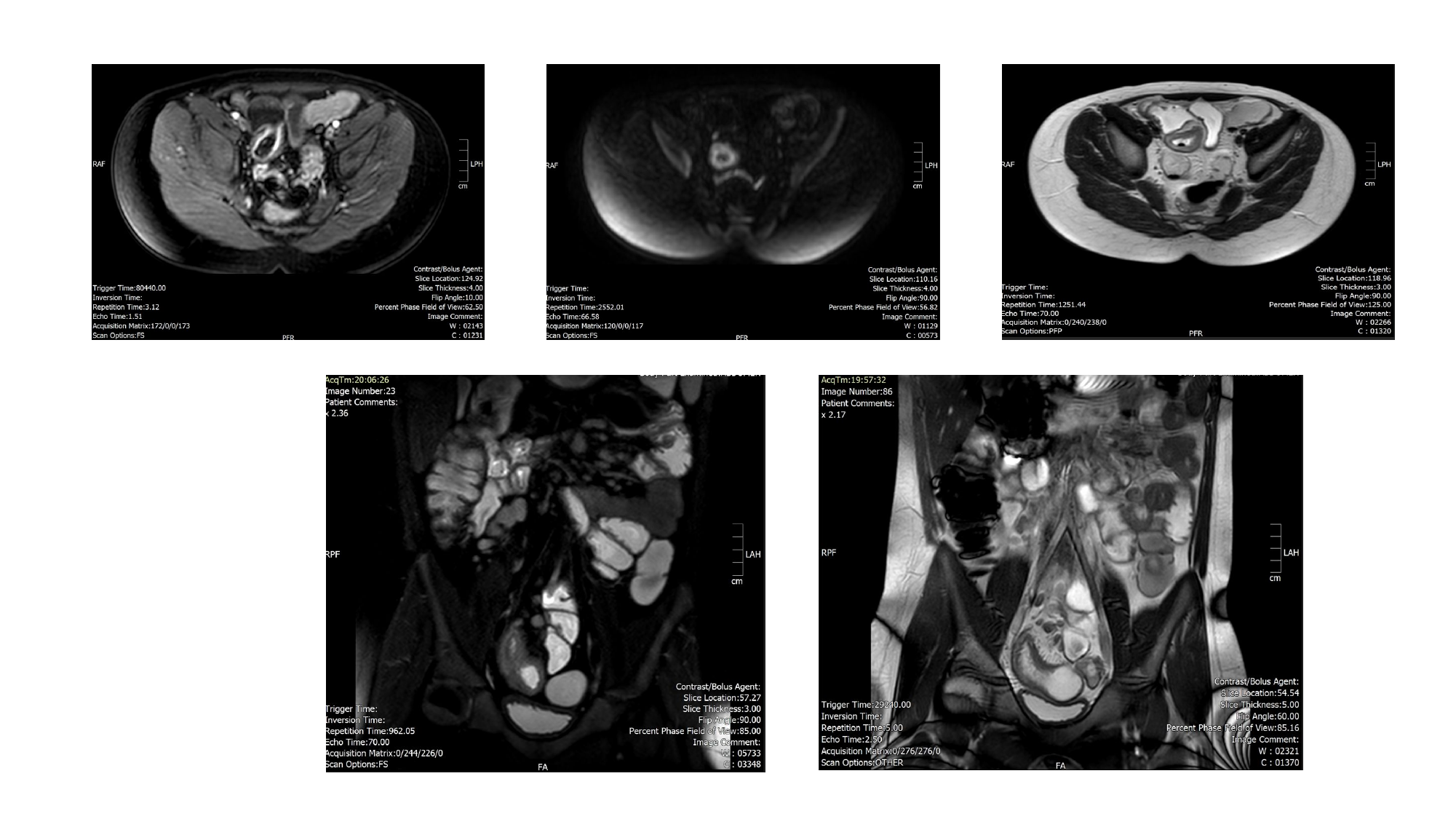

Supplement: Supplementary file 3 — Supplemental Figure 2. Representative scans of one of the patients in our cohort. [file JPN3-82-1451-s001.pptx]
